# Elevated *MTSS1* expression associated with metastasis and poor prognosis of residual hepatitis B-related hepatocellular carcinoma

**DOI:** 10.1186/s13046-016-0361-8

**Published:** 2016-05-26

**Authors:** Xiu-Yan Huang, Zi-Li Huang, Bin Xu, Zi Chen, Thomas Joseph Re, Qi Zheng, Zhao-You Tang, Xin-Yu Huang

**Affiliations:** Department of General Surgery, Shanghai Jiaotong University Affiliated Sixth People’s Hospital, 600 Yi Shan Road, Shanghai, 200233 Peoples Republic of China; Department of Radiology, Xuhui Central Hospital, Shanghai, 200031 Peoples Republic of China; Department of General Surgery, The Tenth People’s Hospital of Tongji University, Shanghai, 200072 Peoples Republic of China; Thayer School of Engineering, Dartmouth College, Hanover, NH 03755 USA; Department of Radiology, Boston Children’s Hospital, Harvard Medical School, Boston, MA 02446 USA; Liver Cancer Institute and Zhongshan Hospital, Fudan University, Shanghai, 200032 Peoples Republic of China

**Keywords:** Metastasis suppressor 1, Hepatocellular carcinoma, Invasion, Metastasis

## Abstract

**Background:**

Hepatectomy generally offers the best chance of long-term survival for patients with hepatocellular carcinoma (HCC). Many studies have shown that hepatectomy accelerates tumor metastasis, but the mechanism remains unclear.

**Methods:**

An orthotopic nude mice model with palliative HCC hepatectomy was performed in this study. Metastasis-related genes in tumor following resection were screened; HCC invasion, metastasis, and some molecular alterations were examined in vivo and in vitro. Clinical significance of key gene mRNA expression was also analyzed.

**Results:**

Metastasis suppressor 1 (*MTSS1*) located in the central position of gene function net of residual HCC. *MTSS1* was up-regulated in residual tumor after palliative resection. In hepatitis B-related HCC patients undergone palliative hepatectomy, those with higher *MTSS1* mRNA expression accompanied by activation of matrix metalloproteinase 2 (MMP2) in residual HCC, had earlier residual HCC detection after hepatectomy and poorer survival when compared to those with lower *MTSS1*. In different cell lines, the levels of *MTSS1* mRNA increased in parallel with metastatic potential. *MTSS1* down regulation via siRNA decreased MMP2 activity, reduced invasive potentials of HCC by 28.9 % in vitro, and averted the deteriorated lung metastatic extent in vivo.

**Conclusions:**

The poor prognosis of hepatitis B-related HCC patients following palliative hepatectomy associates with elevated *MTSS1* mRNA expression; therefore, *MTSS1* may provide a new research field for HCC diagnosis and treatment.

**Electronic supplementary material:**

The online version of this article (doi:10.1186/s13046-016-0361-8) contains supplementary material, which is available to authorized users.

## Background

Liver cancer in men is the fifth most frequently diagnosed cancer worldwide but the second most frequent cause of cancer-related deaths; in women, it is the sixth most commonly diagnosed cancer and the sixth leading cause of cancer-related deaths [1]. Among primary liver cancers, hepatocellular carcinoma (HCC) represents the major histological subtype, accounting for 70 to 85 % of the total liver cancer burden worldwide [2]. Hepatic resection is a standard treatment for patients with HCC. However, even in patients with HCC undergoing curative resection, it is only potentially curative due to the existence of tumor cells or clinically undetectable residual intrahepatic lesions [3]. Our clinical data showed that the proportion of cases with palliative hepatectomy was 34.0 % from 1958 to 2008 (2754/8107, unpublished data). Our follow-up research suggests that HCC patients treated with palliative hepatectomy suffered from dramatically worsened metastases, suggesting that palliative hepatectomy enhances the metastatic potential of residual HCC. Although surgery is associated with tumor suppression and prolonged survival in a few cases [4], increasing number of reports indicate that hepatic resection exacerbates tumor growth and triggers tumor metastasis [5–7]. We found that palliative resection enhances the metastatic potential of residual HCC [5]. The present study sought to explore the underlying molecular mechanism of this metastasis-enhancing effect.

The incidence, development, invasion, and metastasis of HCC are regulated by the activation, inactivation, or dysregulation of several genes. This study employed an orthotopic human HCC model with high metastatic potential in nude mice. This model was developed at Liver Cancer Institute, Fudan University [5, 8]. Using Human Tumor Metastasis Microarray, we screened the metastasis-related genes in tumor tissues following palliative resection, and found that up-regulated metastasis suppressor 1 (*MTSS1*) was located in the central position of gene function net of residual HCC in liver.

*MTSS1* is also known as “missing in metastasis” (*MIM*) gene. Among the multiple *MIM* gene products including MIM-A, MIM-B and MIM-C, MIM-B is the longest and most abundant protein in the cell, which is representative of MIM protein [9]. *MTSS1* has been proposed as a potential metastasis suppressor gene in some studies of HCC [10, 11]. However, other studies have shown that *MTSS1* is highly expressed in various tumors [12, 13], including HCC [14]. *MTSS1* may have an important role in tumor metastasis [12, 15, 16]. *MTSS1/MIM* over-expression is associated with enhanced cell migration, resulting in tumorigenesis, invasion and metastasis [17–19], and predicts poor prognosis in colorectal cancer [20], cervical carcinoma [21], and lung cancer [22]. Recently, Mertz et al. reported that *MTSS1* promotes the metastasis of melanocytes, and high *MTSS1* expression defines a subgroup of primary melanomas with unfavorable prognosis [23]. It remains unclear whether or not *MTSS1* plays a role in metastasis of residual HCC following palliative resection. HCC metastasis involves basement membrane invasion following matrix metalloproteinase (MMP) activation [24]. Previously, we found that palliative resection activates MMP2 in nude mouse models with HCC [5]. In this study, we first screened the metastasis-related genes in residual HCC tissues, and found that *MTSS1* was located in the central position of the tumor gene network. We investigated the *MTSS1* mRNA expression in residual tumor and analyzed its association with prognosis in patients with hepatitis B-related HCC after palliative resection. Subsequently, using in vitro and in vivo studies, we found that *MTSS1* enhanced the invasive and metastatic potential of HCC cells via MMP2 activation. To our knowledge, the current study provides the first evidence that elevated *MTSS1* mRNA expression exacerbates lung metastasis after palliative resection in an HCC model, with poor prognosis of hepatitis B-related patients with HCC treated with palliative hepatectomy.

## Methods

### Patients, specimens and follow-up

The inclusion criteria for patients in this study were (*a*) patients with hepatitis B from 2000 to 2010; (*b*) pathologically proven HCC based on WHO criteria; (*c*) no anticancer treatment prior to first hepatectomy; (*d*) surgical resection twice in 6 months; (*e*) availability of frozen biopsy and/or resected tissues expressing HCC and residual HCC; and (*f*) availability of follow-up data. This study was approved by the Research Ethics Committee of the Shanghai Jiaotong University Affiliated Sixth People’s Hospital, and informed consent was obtained from each patient. HCC patients with hepatectomy were followed up every 2 months during the first postoperative year and at least every 3 to 4 months subsequently, until December 2014 by monitoring serum α-fetoprotein (AFP) levels, abdominal ultrasonography, chest X-ray or computed tomography every 1 to 6 months depending on the patient’s condition. Residual HCC was suspected 2 months after first hepatectomy (HCC-P I group, *n* = 37) in all patients and was confirmed by fine needle autopsy and/or secondary surgery (HCC-P II group, *n* = 37) in 6 months after the first resection. Forty patients with radical hepatectomy (complete resection of tumor with the cut surface free of cancer histologically) and without recurrence at least 12 months after surgery (HCC-R group), and 20 liver tissues from patients with hepatic hemangioma combined with hepatitis B who underwent liver resection were also collected (Liver-T group). All specimens were collected in the operating room immediately (≤15 min) after tissue removal and were snap frozen in liquid nitrogen and stored at −80 °C. General data, metastatic characteristics, pathologic characteristics and survival were compared among the groups.

### Animals, tumor models and cell lines

Five-week-old male athymic BALB/c nu/nu mice weighing 18–20 g each were obtained from the Shanghai Institute of Materia Medica, Chinese Academy of Science. All mice used in these investigations were handled in accordance with the Guide for the Care and Use of Laboratory Animals (National Research Council, 1996), and were approved by the Institutional Animal Care and Use committee of the Shanghai Jiaotong University Affiliated Sixth People’s Hospital. A stepwise metastatic human HCC model system was established in Liver Cancer Institute of Fudan University, which included a metastatic HCC model in nude mice LCI-D20 [8], a HCC cell line MHCC97 with high metastatic potential that originated from LCI-D20 tumor, a high metastatic subclone (MHCC97H with a pulmonary metastatic rate up to 100 % using orthotopic inoculation) and a lower metastatic subclone (MHCC97L with a lung metastatic rate up to 40 % using orthotopic inoculation) established through in vivo selection of MHCC97 [25]. The noninvasive human liver cell line of L02 (normal), human HCC cell lines of Hep3B with very low invasiveness [26], and highly invasive MHCC97H [25], SMCC7721, MHCC97L, HCCLM3 and HCCLM6 were prepared in this study.

### Mice grouping and treatment

Human HCC tumor models produced by MHCC97H were established in nude mice by orthotopic inoculation [8]. Briefly, under anesthesia, the left lobe of the liver was exposed, and part of the liver surface was mechanically injured with scissors. A piece of MHCC97H tumor tissue (size 2 × 2 × 2 mm) was fixed within the liver tissue. Thirty-six nude mice were used in this study.

In the trial experiment, we treated MHCC97H cells with mouse plasma extracted at different time intervals after palliative hepatectomy in nude mice bearing HCC. We found that these cells showed two trans-membrane peaks: one occurring at the 12^th^ hour post-hepatectomy and the other occurring on day 14 post-hepatectomy. In our mouse model, by the day 14 post-hepatectomy, lung metastasis occurred in a majority of the cases. Therefore, we selected the day 14 as a cut-off point to study residual HCC and related lung metastasis, subsequently. Eighteen nude mice bearing HCC xenografts were randomized into three groups 14 daysays after orthotopic implantation: palliative resection group (mice undergoing partial HCC resection with preservation of 2 mm of tumor pedicles), sham operation group (mice with exposed liver but without resection), and blank control group (mice without further surgical intervention). All mice were sacrificed by cervical dislocation 14 days after palliative resection based on pre-experimental results (Additional file 1: Figure. S1A and B). The HCC tissues excised first were designated as tumor tissues T1; tissues excised next from sham operation group were named T2; tissues from blank control group were named tumor tissues T3; and those derived from palliative resection group were denoted T4 that represented residual HCC. Five randomly selected tumor specimens from each group were used for the screening of genes related to metastasis by microarray techniques. Another 18 nude mice treated with palliative resection were randomly injected at multiple points with 100 μL borate-buffered saline (BBS, BBS group), 2 × 10^7^ U Lenti-GFP (Lenti-GFP group) or Lenti-MTSS1 (Lenti-MTSS1 group), respectively, under ultrasound guidance, and the procedure was repeated 3 h later. All mice were sacrificed by cervical dislocation 14 d post-resection for the evaluation of early metastasis.

### Parameters, sample preparations and grading of lung metastasis

Mice were sacrificed for gene profiling of pulmonary metastatic nodules. Lung tissues, tumor tissues, mRNA and protein extracts were harvested for further studies. Serial sections of lung tissue measuring 5 μm in thickness were obtained, and all metastases were verified histologically. The degree of lung metastasis was graded by the maximum number of tumor cells counted in the solitary pulmonary metastatic nodules (grades): grade I, < 20; grade II, 20–50; grade III, 50–100; and grade IV, > 100 [27].

### Tumor metastasis-related gene profiling

In recent years, more and more researches employed Human Tumor Metastasis Microarray to dissect the gene function and form gene regulatory network in various of cancers [28–30]. Thus, the Human Tumor Metastasis Microarray is useful in searching for new therapeutic targeted genes and helpful in dissect mechanism of the gene in several of cancers [31, 32]. The OHS-028 OligoGEArray Human Tumor Metastasis Microarray (Super Array, Bethesda, MD) was used to characterize the metastasis-related gene expression profiles of HCC tissues in this study. GEArray Expression Analysis Suitesoft ware was used for gene analysis. The methods of Support Vector Machine (SVM), gene significance analysis and gene correlation degree analysis were adopted to detect the potential markers involved in HCC metastasis. To further explore the relationship between gene function and gene function, we built the GO-network (gene function net). In the GO-network, the degree represented the relationship between GO terms. The key GO terms in the network were signal transduction, multicellular organismal development, ion transport, and signal transduction in the GO terms. Among them, multicellular organismal development, GO terms had the highest degree in the network. Then, a gene function net was constructed based on specific gene clustering analysis and multi-dimensional scale [33, 34].

This study describes the advantages of using Support Vector Machines algorithm for the classification of gene expression data. Using the RVM model, we found the different genes including the *d* genes from microarray data. Our conclusions are as follows. The characteristic of samples, which amount to *n* was allocated the different gene set, and we obtain a matrix (*X*)_*n* × *d*_ from the samples. The liver cancers samples, which are high or low, constituted the dataset of {*x*_*i*_, *y*_*i*_}(*i* = 1, 2, ⋯, *n*), where the *x*_*i*_ represents the *d* dimension vector, and *y*_*i*_ ∈ {*high* = 1, *low* = − 1} is the class label. We obtained an optimal hyperplane derived from the SVM algorithm. The optimal hyperplane that is high and low is as follows: Minimizing $\frac{1}{2}{\left\Vert \omega \right\Vert}^2$ and Subject to *y*_*i*_ × 〈*x*_*i*_, *ω*〉 + *b* ≥ 1. From the quadratic form, we selected the Support Vectors which carried weights, and decision functions as follows: ${\displaystyle \overset{\wedge }{f(x)}}= sign\left(\left\langle {\omega}^0,X\right\rangle +b\right)= sign\left({\displaystyle \sum_{SVs}{\alpha}_i{y}_i\left\langle {x}_i,X\right\rangle }+{b}^0\right)$. We transformed the nonlinear Microarray data into linear format using the gauss and ploy kernel functions. Gauss kernel function was as follows: $k\left({x}_i,{x}_j\right)= \exp \left(\frac{-{\left\Vert {x}_i-{x}_j\right\Vert}^2}{\sigma^2}\right)=\left\langle \varPhi \left({x}_i\right),\varPhi \left({x}_j\right)\right\rangle$. The ploy kernel function was as follows: *k*(*x*_*i*_, *x*_*j*_) = (〈*x*_*i*_^*T*^, *x*_*j*_〉 + 1)^*d*^ = 〈*Φ*(*x*_*i*_), *Φ*(*x*_*j*_)〉, where 〈*Φ*(*x*_*i*_), *Φ*(*x*_*j*_)〉 is a scalar product. Because the {*x*_1_, *x*_2_, ⋯, *x*_*n*_} represents nonlinear space, we transformed the vector *x*_*i*_ into high-dimensional feature space using the kernel function. The prediction function is as follows: ${\displaystyle \overset{\wedge }{f(x)}}= sign\left(\left\langle {\omega}^0,\varPhi (X)\right\rangle +b\right)= sign\left({\displaystyle \sum_{SVs}{\alpha}_i{y}_i\left\langle \varPhi \left({x}_i\right),\varPhi (X)\right\rangle }+{b}^0\right)$. We demonstrated that the gauss kernel function was effective with a predictive accuracy close to 90 %.

The expression datasets presented in this publication have been deposited in NCBIs Gene Expression Omnibus (http://www.ncbi.nlm.nih.gov/geo/) and are accessible through GEO Series accession number GSE75965.

### Hematoxylin and eosin (H&E) stains

Paraffin blocks of 10 % buffered formalin-fixed samples of tumor and lung tissues were prepared, and 5–μm-thick serial sections were obtained. Pulmonary metastatic nodules were verified via Hematoxylin and Eosin (H&E) stains.

### Real-time fluorescent quantitative polymerase chain reaction (Real-time PCR)

The primers used for real-time polymerase chain reaction (PCR) were designed using Primer Premier 5.0 (Premier Biosoft International, Palo Alto, CA): human *MTSS1* forward, 5′-tagctggaaggactgggcta-3′, and reverse, 5′-agtcatgctccgtggtctct-3′. *GAPDH* forward and reverse primers were 5′-ggtgaaggtcggagtcaacg-3′ and 5′-accatgtagttgaggtcaatgaagg-3′, respectively. PCR was performed in the Rotor-Gene 3000 PCR system (Corbett Research, Sydney, Australia). Conditions for PCR were 37 °C for 2 min, 94 °C for 3 min, 40 cycles for *GAPDH* of 94 °C for 5 s, 60 °C for 40 s, followed by 37 °C for 5 s, and 95 °C for 30 s, 95 °C for 30 s, 40 cycles for *MTSS1* of 95 °C for 15 s, 60 °C for 15 s, 72 °C for 30 s. Finally, baseline and threshold values of these genes were set using the Rotor-Gene 6.0 (Corbett Research) for analysis.

### Western blot

Proteins were separated by 10 % sodium dodecyl sulfate -polyacrylamide gel electrophoresis and transferred onto polyvinylidene difluoride membranes (Millipore, Bedford, MA). The membrane was blocked with 5 % non-fat dried milk in TBST (20 mM Tris–HCl, 150 mM NaCl, and 0.1 % Tween 20, pH 7.5) for 2 h and incubated overnight with antibodies against *MTSS1* (Abnova, Caltag-Medsystems Ltd., Buckingham, UK) at 4 °C. After washing with TBST buffer, membranes were incubated with horseradish peroxidase-conjugated anti-mouse IgG secondary antibodies for 1 h at room temperature and detected by enhanced chemiluminescence detection system (Amersham-Pharmacia Biotech, Braunschweig, Germany). *GAPDH* was used as an internal control (Santa-Cruz Biotechnologies, California, USA).

### siRNA design and lentivirus construction

The small interfering RNA (siRNA) sequences targeting *MTSS1* were designed according to a modified Tuschel standard, and the detailed sequences were as follows:

Target 1:

(+) 5′-CGGCCAGTGATTGAAGAAGAA-3′,

(−) 5′-TTCTTCTTCAATCACTGGCCG-3′;

Target 2:

(+) 5′-GCTGGATAAAGACCACGCAAA-3′,

(−) 5′-TTTGCGTGGTCTTTATCCAGC-3′;

Target 3:

(+) 5′-CCTTCCAGACTACGCTCATTA-3′,

(−) 5′-TAATGAGCGTAGTCTGGAAGG-3′;

Target 4:

(+) 5′-CCCATGACTCAGGATTCATAT-3′,

(−) 5′-ATATGAATCCTGAGTCATGGG-3′

and Target 5:

(+) 5′-GACCATCTCGGAAGATCTAAA-3′,

(−) 5′-TTTAGATCTTCCGAGATGGTC-3′.

The target 2 sequences were selected for further study. The siDNA sequences matching the designed siRNA were then amplified with PCR and inserted into a mouse U6 promoter-driven lentivirus plasmid (purchased from Genechem Co., Shanghai, China), in which a luciferase expressing construct was then incorporated, forming the pGCL-MTSS1-GFP plasmid. In brief, the infectious titer was determined by fluorescence-activated cell sorting analysis of GFP-positive in 293 T cells. The virus titers were in the range of 10^7^ transducing units/mL medium.

### Stable lentiviral transfection of HCC cells

HCC cells were plated in 24-well plates. Subconfluent cells were infected with 3 mL/well three times (about 3 h per infection). The cells were divided into three groups: the knockdown cells transfected with *MTSS1* siRNA lentivirus (KD group), the negative control cells transfected with empty lentivirus (NC group) and the blank control cells not transfected (CON group).

### *MTSS1* overexpression

The full sequence of *MTSS1* was amplified by the standard PCR procedure. The primer sequences for cloning include sense primer: ATGGAGGCTGTGATTGAG; and antisense primer: CTAAGAAAAGCGAGGGG. We transfected the pCMV6-empty and pCMV6-MTSS1 into Hep3B cancer cells and RNA and protein were extracted. *MTSS1* overexpression was confirmed, and invasive potential assessed.

### In vitro invasion assay

The MHCC97H cells (5 × 10^4^ cells/well) treated with *MTSS1* siRNA lentivirus, empty lentivirus or with control (no treatment) were added to the upper chamber (100 μL DMEM), and 600 μL conditioned medium was added to the lower chamber. After 24 h of incubation, the invaded cells were fixed with methanol and stained with crystal violet solution. The results were expressed as the number of penetrated cells under a microscope at 200× magnification in five random fields and presented as the means ± SD of three assays.

### Gelatin zymography assay

Zymography for MMP2 activity was conducted via sodium dodecyl sulfate-polyacrylamide gel electrophoresis (SDS-PAGE) [35], with modification. The MHCC97H cells (5 × 10^4^ cells/well) treated with or without *MTSS1* siRNA lentivirus were serum-starved for 24 h before cell supernatant collection. The molecular weights of these bands indicating MMP2 activity were determined by molecular weight standards (Bio-Rad Laboratories, Hercules, CA). The assays were conducted in triplicate.

### MMP2 activity assay

Human MMP2 activity was quantified with an enzyme-linked immunosorbent assay (ELISA) System (Amersham Pharmacia Biotech, Piscataway, NJ) according to the manufacturer’s instructions. The plate was read at 450 nm in a SPECTRAmax 250 Microplate Spectrophotometer (Molecular Devices, Sunnyvale, CA). The assays were conducted in triplicate.

### Statistical analysis

All continuous variables were expressed as means ± SD or means ± SE. ANOVAs, Student *t* test and Cochran-Mantel-Haenszel (CMH) test (Row Mean Scores Differ) were used for statistical comparison among groups. *MTSS1* expression and clinicopathological parameters were analyzed by Pearson chi-square test or Fisher’s exact test. The survival analysis was conducted using the Kaplan-Meier method. The statistical SAS software package (Version 8.2, SAS Institute, Inc., Cary, NC, USA) was adopted for data analysis, and the statistical significance was defined as *P* < 0.05.

## Results

### *MTSS1* located in the central position of the gene function net of residual HCC

**Fig. 1:**
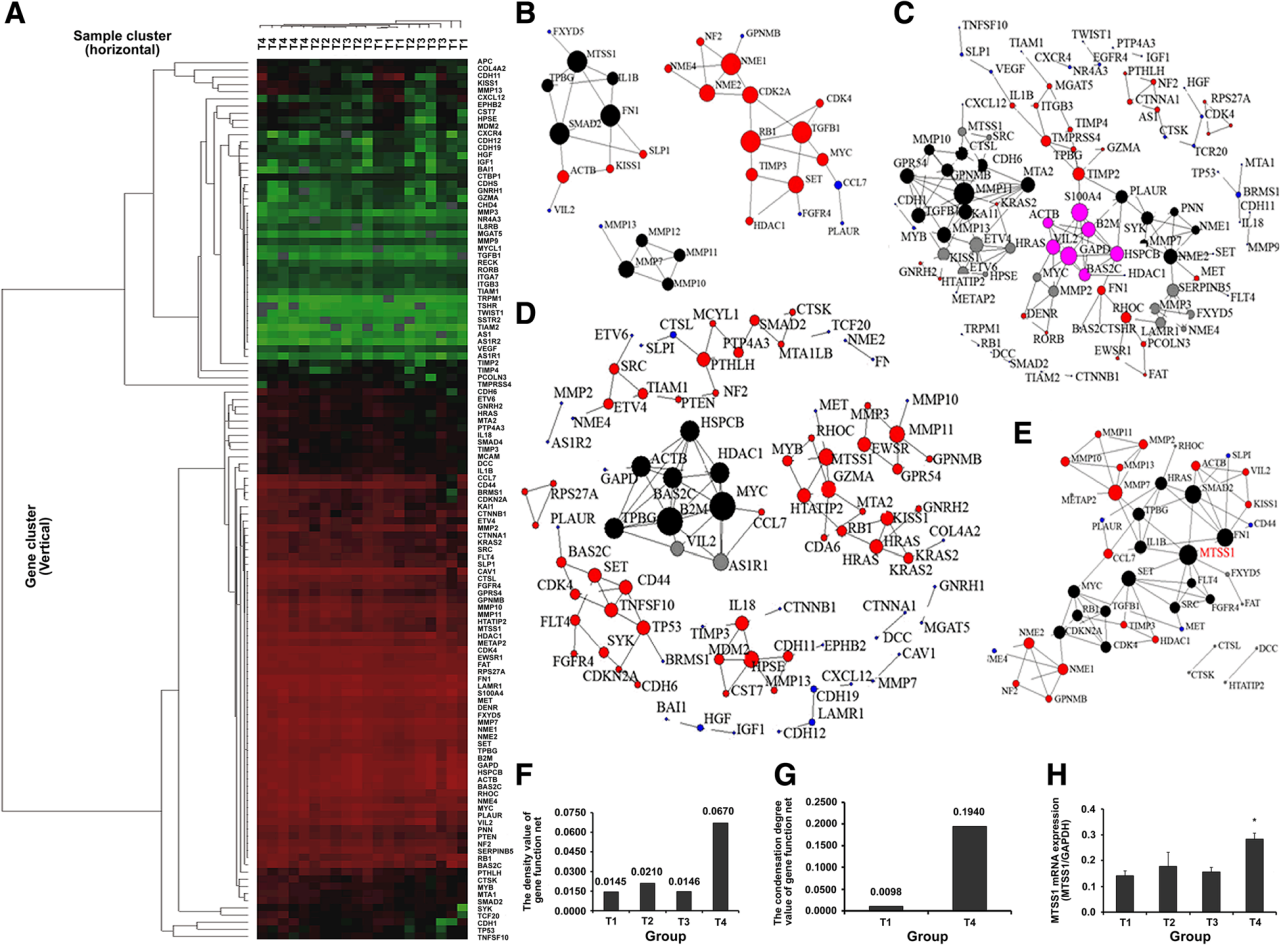
*MTSS1* located in the central position of the gene function net of residual HCC. **a** the cluster heat map of gene cluster (vertical) and sample cluster (horizontal) were based on tumor-related genes in HCC. The color red and green represents high and low gene expression, respectively; samples with expression above mean level (red) are clustered. Gene function nets of HCC were constructed successfully. **b** gene function nets of HCC from palliative resection group (HCC tissues T1). **c** gene function nets of HCC from sham operation group (HCC tissues T2). **d** gene function nets of HCC from blank control group (HCC tissues T3). **e** gene function nets shown that *MTSS1* was located in the central position of the gene function net of residual HCC (HCC tissues T4). **f** the density value of the gene function net. **g** the condensation degree of the net. **h** qRT-PCR analysis shown that palliative resection up-regulated *MTSS1* expression, *P = 0.001, 0.034, and 0.002, respectively, when compared with controls (T1, T2, and T3 groups). The bars indicate the means ± s.d

Palliative HCC resection promoted tumor metastasis. Therefore, we first screened the metastasis-related genes in HCC tissues to detect the altered genes in residual tumor. Gene cluster and sample cluster analysis were based on tumor-related genes found in the HCC tissues (Fig. 1a). Gene function nets were constructed successfully (Fig. 1b, c, d and e). *MTSS1* was located in the central position of the gene function net of residual HCC (Fig. 1e). Analysis of differential gene expression showed that the density of the net (Fig. 1f), which reflects the relevance of different genes, was higher in the palliative group (T4 group, 0.0670) than in controls (T1 group, 0.0145; T2 group, 0.0210; T3 group, 0.0146), and the condensation degree of the net (Fig. 1g) in residual HCC (T4 group, 0.1940) was substantially higher than in the corresponding control group (T1 group, 0.0098). *MTSS1* mRNA expression (Fig. 1h) was up-regulated in residual HCC (T4 group) when compared with controls (T1, T2, and T3 groups, *P* = 0.001, 0.034, and 0.002, respectively).

### Elevated *MTSS1* mRNA expression is associated with poor prognosis

**Fig. 2:**
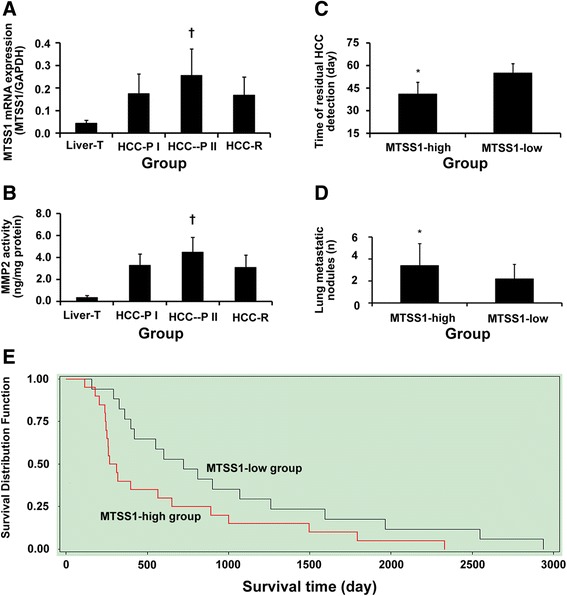
Elevated *MTSS1* mRNA expression is associated with poor prognosis. **a** the qRT-PCR analysis of *MTSS1* expression in different groups, *P* = 0.000, when compared with controls. **b** MMP2 activity in residual HCC (HCC-P II group); †*P* = 0.000, when compared with controls; no difference in *MTSS1* mRNA level or MMP2 activity between HCC-P I and HCC-R groups. **c** earlier detection of residual HCC in MTSS1-high group than in MTSS1-low group, *P = 0.000. **d** higher number of lung metastatic nodules in MTSS1-high group than in MTSS1-low group, *P = 0.039, when compared with controls. **e** longer survival in MTSS1-low group compared with MTSS1-high group, *P* = 0.023

We found that *MTSS1* was situated in the central position of gene function net of residual HCC in the nude mice model. We tested *MTSS1* expression in tumor tissues from HCC patients. HCC tissues expressed higher *MTSS1* mRNA level than the control liver tissues (all *P* = 0.000, Fig. 2a). *MTSS1* mRNA level was significantly increased in residual HCC tissues (HCC-P II group) compared with controls (all *P* = 0.000, Fig. 2a). In the meantime, MMP2 was also significantly activated (*P* = 0.000, Fig. 2b). There was no difference in *MTSS*1 mRNA level or MMP2 activity between tissues derived from HCC-P I and HCC-R groups.

The mean *MTSS1/GADPH* mRNA level of 0.18 was calculated out in the HCC-P I group. Then, we split the patients following palliative resection by *MTSS1* expression in residual HCC: > 0.18 (MTSS1-high group, *n* = 20); and ≤ 0.18 (MTSS1-low group, *n* = 17). Correlation of *MTSS1* mRNA expression with their clinicopathological parameters is displayed in Table 1. A higher level of *MTSS1* mRNA was significantly correlated with tumor number (*P* = 0.035), presence of HCC satellite (*P* = 0.019), incomplete or no encapsulation (*P* = 0.008), presence of vascular invasion (*P* = 0.042) and advanced TNM stage (*P* = 0.033). Further, most of the AFP-negative patients (9 of 11, 81.8 %) showed higher *MTSS1* mRNA levels (*P* = 0.036). No significant association between *MTSS1* mRNA and age, sex, liver cirrhosis, tumor size, or γ-glutamyl transferase was found.

**Table 1:** Correlation of tumor *MTSS1* mRNA expression level with clinicopathological parameters of HCC patients

Clinicopathological parameters	Number	*MTSS1/GAPDH* mRNA (*n*)		*P*
		MTSS1-low group	MTSS1-high group	
		(≤0.18, *n* = 17)	(>0.18, *n* = 20)	
Age	37			0.900
≤ 52 y	20	9	11	
> 52 y	17	8	9	
Sex	37			0.588
Female	8	3	5	
Male	29	14	15	
Cirrhosis	37			0.855
Yes	30	14	16	
No	7	3	4	
Tumor number	37			0.035*
Multiple	20	6	14	
Single	17	11	6	
Satellite	36^a^			0.019*
Yes	15	3	12	
No	21	13	8	
Tumor size, cm	37			0.212
≤ 5	7	5	2	
> 5	30	12	18	
Encapsulation	37			0.008**
Complete	15	11	4	
Incomplete or none	22	6	16	
Vascular invasion	36^a^			0.042*
Yes	21	6	15	
No	15	11	4	
TNM stage	37			0.033^*^
Early stage (II)	7	6	1	
Advanced stage (III-IV_A_)	30	11	19	
AFP level, ng/mL	37			0.036*
≤ 20	11	2	9	
> 20	26	15	11	
γ-Glutamyl transferase, units/L	37			0.134
≤ 54	19	11	8	
> 54	18	6	12	
Adjuvant TACE	37			0.815
Yes	16	7	9	
No	21	10	11	

*NOTE*: *HCC* hepatocellular carcinoma, *TACE* transcatheter arterial chemoembolization

Significant difference: * < 0.05, ** < 0.01

^a^Number less than 37 due to missing data

Patients with higher *MTSS1* mRNA expression revealed earlier residual HCC (*P* = 0.000, Fig. 2c), a higher number of lung metastatic nodules (*P* = 0.039, Fig. 2d), and lower 1-year survival rate after the first resection [40.0 % (8/20) vs. 76.5 % (13/17), *P* = 0.026]. The survival of the MTSS1-high group was significantly shorter than that of the MTSS1-low group (*P* = 0.023, Fig. 2e). In the MTSS1-high group, when sub-grouping patients according to the mean value of *MTSS1/GADPH* mRNA expression (0.33, *n* = 20), only 1 out of 9 in those with *MTSS1/GADPH* mRNA higher than 0.33 survived for more than 1 year, whereas 7 out of 11 with *MTSS1/GADPH* mRNA lower than 0.33 survived for more than 1 year (*P* = 0.028). There was no significant difference in the 3-year or 5-year survival rates.

The cumulative survival rates were 56.8 % (21/37) for 1 year and 21.6 % (8/37) for 3 years. The 5-year survival in these patients was 10.8 % (4/37), which was lower than that of all patients with palliative resection performed in our hospital between the years 1958 and 2008 (30.0 %, 826/2754, *P* = 0.010, unpublished). None of the 37 patients in the current study survived longer than 10 years. Transcatheter arterial chemoembolization (TACE) did not significantly affect *MTSS1* mRNA expression and survival between groups or subgroups.

### Altered *MTSS1* expression is correlated with metastatic potential

**Fig. 3:**
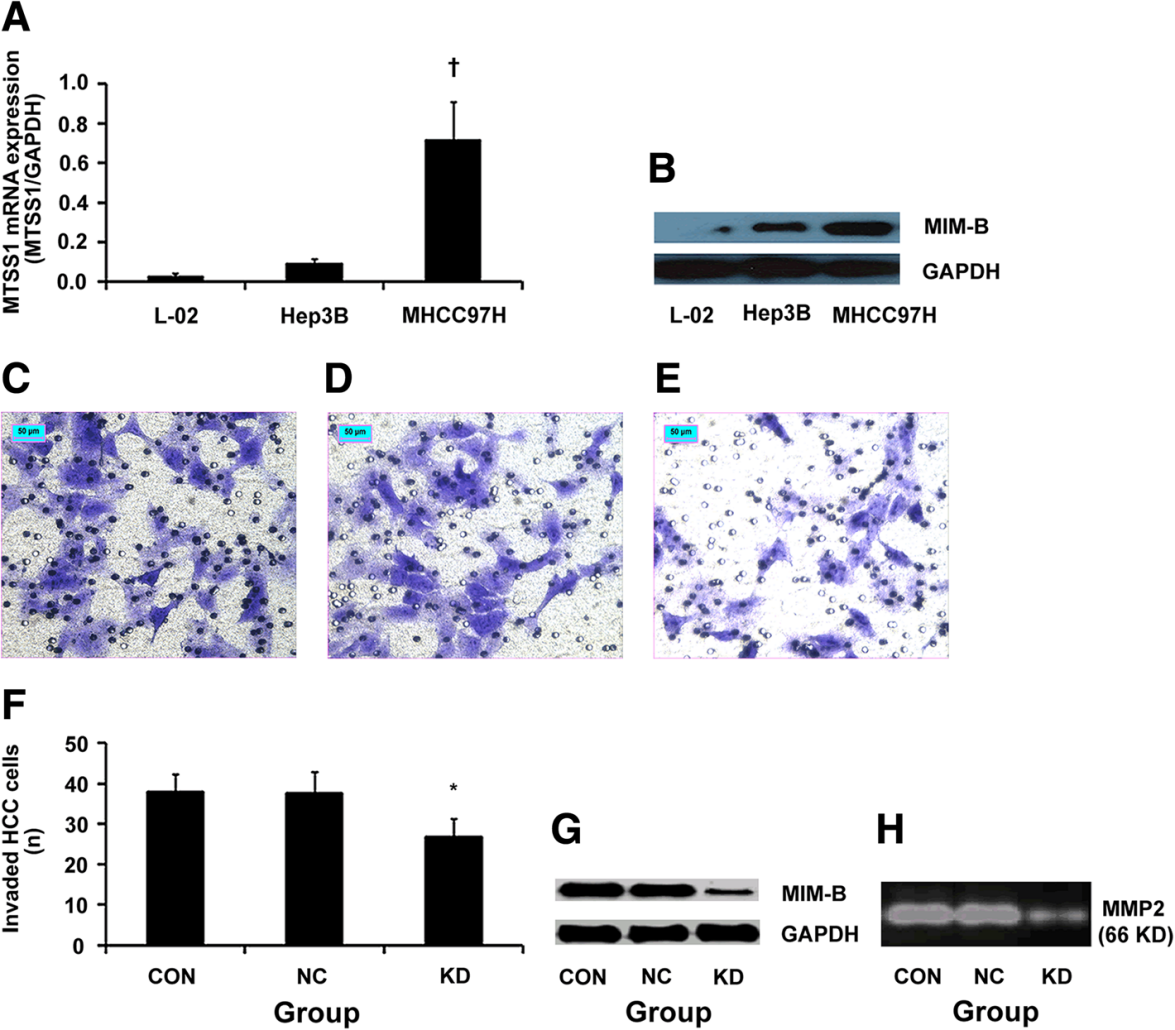
*MTSS1/*MIM-B expression in different cell lines. **a**, qRT-PCR analysis of *MTSS1* mRNA expression; †*P* = 0.000, 0.001, respectively, when compared with cell lines of L02 and Hep3B. **b**, western blot analysis of MIM-B protein level; the bars indicate the means ± s.d. Stable lentiviral transfection of MHCC97H cells with siRNA targeting *MTSS1*, ×200, *bar*, 50 μm. Invasion assay analysis shown that the number of transfected HCC cells (original magnification, × 200) after treatment with *MTSS1* siRNA lentivirus (**e**) was lower than that of controls (**c**, **d**), *bar*, 50 μm*.* **f**, the difference in invasiveness, *P = 0.000, when compared with controls. The assays were conducted in triplicate. The bars indicate the means ± s.d. **g**, western blot analysis demonstrated that MIM-B was down-regulated by siRNA. **h**, results of gelatin zymography assay shown that MMP2 activity was inhibited (0.6-fold) by siRNA, *P* = 0.000, compared with controls

To identify the relationship between *MTSS1* expression and metastatic potential of HCC, we examined the *MTSS1* expression in different cell lines. Significant differences in *MTSS1* mRNA levels were found between the L02 cell line without metastatic potential, the Hep3B cell line with very low metastatic potential, and the MHCC97H cell line with higher metastatic potential (*P* = 0.000, 0.001, respectively, Fig. 3a). The metastatic potential of cell lines increased in parallel with increased levels of MIM-B protein, encoded by *MTSS1* (Fig. 3b).

The siRNA targeting *MTSS1* was constructed successfully with high transfection efficiency, which markedly inhibited the invasive ability of MHCC97H cells by 28.9 % (Fig. 3c, d and e). The number of invaded HCC cells in *MTSS1* knockdown group (KD group) was less than in the empty lentivirus group (NC group) and the blank control group (CON group, all *P* = 0.000, Fig. 3f). The MIM-B protein level in KD group was down-regulated when compared with controls (Fig. 3g). Gelatin zymography assay showed that MMP2 activity was decreased significantly (0.6-fold, *P* = 0.000, Fig. 3h). Further, in vitro studies showed that *MTSS1* overexpression in Hep3B cells promoted cell invasion by 18.2 % (*P* = 0.021, Additional file 1: Figure. S2A and B). The effects of *MTSS1* downregulation and overexpression in HCC cells were validated by several other HCC cell lines. *MTSS1* downregulation inhibited the invasive ability of HCCLM3 and HCCLM6 cells by 25.7 % (*P* = 0.002) and 23.1 % (*P* = 0.001), respectively, while *MTSS1* upregulation increased the invasive ability of SMCC7721 and MHCC97L cells by 14.8 % (*P* = 0.017) and 12.5 % (*P* = 0.011), respectively.

### SiRNA targeting *MTSS1* attenuates pulmonary metastasis following palliative resection in nude mice bearing human HCC xenograft

**Fig. 4:**
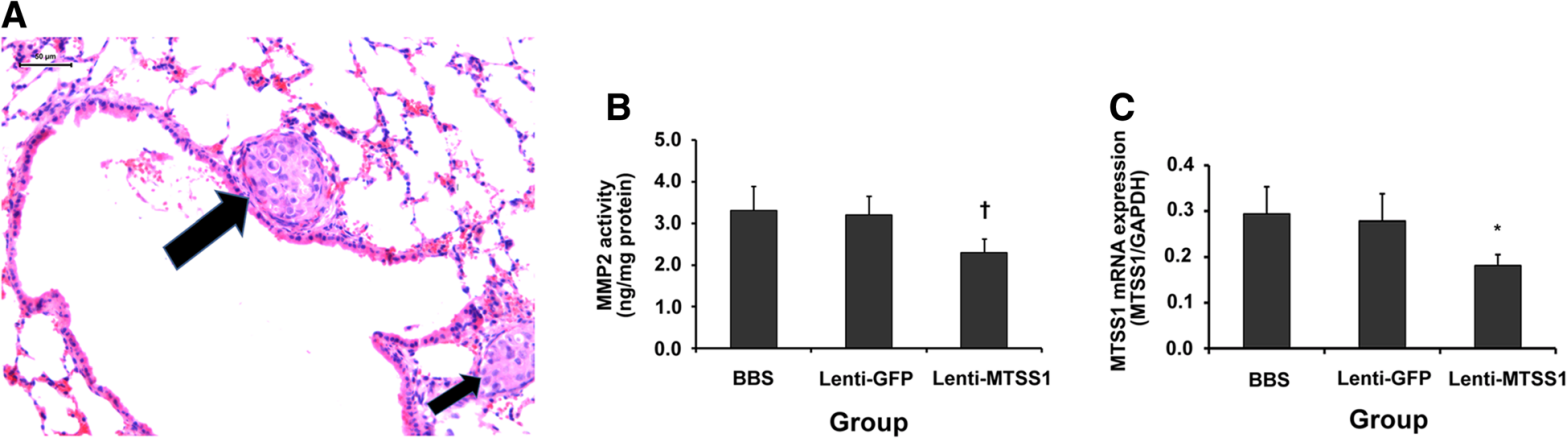
SiRNA targeting *MTSS1* attenuates pulmonary metastasis following palliative resection in nude mice bearing human HCC xenograft. **a**, two pulmonary metastatic nodules (*arrows*, original magnification, × 200), *bar*, 50 μm. **b**, MMP2 activity was inhibited by siRNA targeting *MTSS1* in vivo, †*P* = 0.003 compared with controls. **c**, qRT-PCR analysis demonstrated that *MTSS1* expression was inhibited by siRNA in vivo, *P = 0.039 compared with controls. The bars indicate the means ± s.d

Subsequently, we used in vivo studies to validate the in vitro effects of siRNA targeting *MTSS1*. Two weeks following palliative HCC resection, the lung metastasis (Fig. 4a) was upgraded when compared with controls (*P* = 0.039, Table 2). In Lenti-MTSS1 group, the pulmonary metastasis was downgraded after *MTSS1* knock down (*P* = 0.041, Table 2). MMP2 activity of the residual HCC was also significantly inhibited along with *MTSS1* inhibition in the Lenti-MTSS1 group when compared with controls (*P* = 0.003, 0.039, respectively, Fig. 4b and c). Further, no animal experienced therapy-related side effects following siRNA administration.

**Table 2:** Effects of palliative resection and Lenti-MTSS1 on pulmonary metastasis

Group	The total number of solitary pulmonary nodule	Lung metastatic extent (Grade)*		
	(*n*)	I	II	III-IV
Palliative Resection	35	12	11	12
Sham Operation	33	19	8	6
Black Control	32	20	7	5
BBS	32	9	10	13
Lenti-GFP	31	8	11	12
Lenti-MTSS1	30	15	10	5

*NOTE*: All pulmonary metastases were verified via histological examination when mice were sacrificed 2 weeks following palliative resection, or receiving BBS, Lenti-GFP, or Lenti-MTSS1. Lung metastatic extent was graded by number of tumor cells counted in the maximum section of the solitary pulmonary metastatic nodule. *P = 0.039 or 0.041 when Palliative Resection group or Lenti-MTSS1 group was compared among Control groups, respectively

## Discussion

HCC is one of the deadliest types of cancers, with a mortality of almost 100 % [36]. The mortality of HCC remains high because the disease is typically diagnosed when it is already at an advanced stage, when most potential curative therapies such as resection and transplantation are of limited efficacy. Recent studies reported that different therapies including surgery [5–7], hepatic artery ligation [37], insufficient radiofrequency ablation [38–40], and sublethal heat treatment [41] promoted residual tumor progression. It is especially important to investigate the underlying mechanism. Our research team has developed a safe and reliable method of palliative HCC resection in an orthotopic nude mouse model, and reported that palliative resection enhances metastatic potential of residual HCC in liver [5]. In the present study, using this method, we investigated the precise mechanism mediating this metastasis.

Data from our institution (1958–2008, unpublished) revealed that in HCC patients who underwent hepatectomy the proportion of cases with palliative HCC resection was 34.0 % (2754/8107). The 5-year survival of patients who underwent palliative HCC resection (30.0 %, *n* = 2754) was much lower than in those who underwent curative resection (52.6 %, *n* = 5353). It suggested that HCC palliative resection may promote HCC aggressive metastasis in residual tumor. Using gene microarray techniques and bioinformatics tools, we first found that *MTSS1* was upregulated and situated in the central position of gene function net of residual HCC in nude mouse models. We tested the *MTSS1* expression in HCC tissues from HCC patients who underwent palliative hepatectomy. We found that patients with high *MTSS1* expression manifested residual liver tumors early, higher number of metastatic lung nodules and lower 1-year survival rates, which suggests that HCC patients with high *MTSS1* expression have a poor prognosis and a higher mortality following palliative hepatectomy. No significant changes in the survival rates were detected between high- and low-MTSS1 groups after 3 or 5 years, which might be an artifact of the small sample size as few patients survived beyond the first year, especially in high-MTSS1 high patients. Notably, the *MTSS1* levels were higher in cancers at advanced stage than in early stage, in our study. In contrast, Ma et al. reported that patients with early pTNM stage (I-II) manifested high *MIM-B* mRNA expression [14]. Consistent with our study, other studies reported that the positive expression rates of *MTSS1* were significantly higher in advanced stages of colorectal cancer [20] and cervical carcinoma [21]. Interestingly, most AFP negative patients (9 of 11, 81.8 %) showed higher *MTSS1* mRNA levels in this study, suggesting that *MTSS1* may be another early predictor of residual HCC, which merits investigation with a large group of patients.

The cumulative 5-years-survival rate in this group of 37 cases (10.8 %, 4/37) was lower than in all patients undergoing palliative resection at our hospital during 1958 – 2008 (30.0 %, 826/2754). The reasons may be related partially to reoperation 37 cases. In another group of 2754 cases, patients underwent reoperation, TACE or other therapeutic methods, which suggested that palliative reoperation decreases 5-years-survival rate. Therefore, we may increase the 5-years-survival rate with reasonable therapeutic care of patients undergoing palliative resection. However, this hypothesis requires further investigation in a larger number of cases.

Our in vitro studies found that the *MTSS1/*MIM-B expression was increased in parallel with the metastatic potential of cell lines. Downregulation of *MTSS1* reduced the invasive potential of HCC cells and prevented the exacerbated of lung metastasis following palliative resection in nude mice bearing HCC xenografts. *MTSS1/*MIM-B is also found upregulated in the early stages of several cancers, including melanomas [42], head and neck squamous cell carcinoma [43], and lung cancer [22]. *MIM-B* mRNA and protein is also proved overexpressed in HCC [14]. Therefore, we believe that advanced lung metastasis after palliative surgery may correlate with upregulated *MTSS1* expression.

In our study, patients had chronic hepatitis B infection which progressed to HCC, and we found that the poor prognosis of hepatitis B-related HCC patients following palliative hepatectomy associates with elevated *MTSS1* mRNA expression. Thus, to dissect the regulation of *MTSS1* expression in HCC may provide a new research field for HCC diagnosis and treatment. Previous study demonstrated that p63 could control the cell migration and invasion by regulating the *MTSS1* expression in breast tumor cells [44]. Recent reports revealed that chronic hepatitis B infection could induce gene expression in several of cancers [45, 46]. Therefore, there is a possibility that the virus infection plays some role in the expression of *MTSS1*, which needs further study to identify the role of chronic hepatitis B infection in *MTSS1* regulation in HCC. Furthermore, p63 may affect cell migration and invasion by regulating the *MTSS1* expression in HCC that remains to be fully elucidated.

The mechanism of *MTSS1* upregulation in HCC after palliative surgery is unclear. The altered microenvironment may be a key player. Predina et al. reported the absence of any changes in the tumor cells per se but rather in the surrounding tumor microenvironment after surgery [47]. Surgery generates a sequence of events to induce wound healing and is characterized by the release of VEGF, PDGF, prostaglandins, TGF-beta, clotting factors, and complement [47–49], which may contribute to the high levels of *MTSS1* by as unknown mechanism.

Cancer metastasis involves basement membrane invasion by MMP activation [50]. A higher level of MMP2 has been related to a poorer prognosis in HCC patients [35, 51–53]. Our results revealed that *MTSS1* expression, MMP2 activity and lung metastasis were increased after palliative hepatectomy both in nude mice models and in HCC patients. After down-regulating *MTSS1* expression, the MMP2 activity and lung metastasis were also decreased. The synchronous alteration of MMP2 with *MTSS1* suggests that *MTSS1* promotes tumor metastasis by targeting MMP2. Based on our in vitro and in vivo studies, we believe that the metastasis-enhancing effect of palliative resection may partially be due to the dysregulation of the MIM-B/MMP2 pathway. We found MMP2 was activated after palliative HCC resection [5]. However, the mechanism underlying *MTSS1*-mediated MMP2 activation is still unclear. Further, *MTSS1* encodes an intracellular MIM that is implicated in actin cytoskeletal reorganization, and *MTSS1* represents a novel signaling pathway from PDGF receptor to the actin cytoskeleton via Src-related kinases [54]. Therefore, *MTSS1* depletion affects the cytoskeletal reorganization. Giacobbe et al. reported that *MTSS1* enhances breast tumor cell migration resulting in poor prognosis [44]. We concluded that *MTSS1* depletion affects the migratory capabilities of HCC cells, which needs further investigation.

Currently, there are several limitations in - and resistance to the therapy of HCC, thus, there is a necessary need for development of new and more effective therapeutic alternatives [55, 56]. In this study, patients with increased *MTSS1* expression in residual HCC showed the worst prognosis. Downregulation of *MTSS1* successfully averted pulmonary metastasis in our mouse model. The findings suggest that individualized treatment targeting high *MTSS1* expression may be an effective strategy to treat HCC.

There are some limitations in this study. The nature of the nude mice lowers the strength of evidence. The size of our patient population is relatively small, which may lead to selection bias. Finally, we cannot exclude the possibility that other downstream pathways of *MTSS1* might also mediate HCC metastasis after palliative hepatectomy. Therefore, the precise mechanism triggering metastasis remains to be fully elucidated. Despite these limitations, we believe that the current study provides preliminary and powerful data underscoring the value of *MTSS1/*MIM-B expression in HCC diagnosis and treatment.

## Conclusions

In summary, palliative HCC resection upregulates *MTSS1* mRNA expression, activates MMP2 activation and enhances residual HCC metastasis to lung. Patients with hepatitis B-related HCC exhibiting high *MTSS1* mRNA levels in the residual tumor show poor prognosis after hepatectomy. However, additional investigations into the mechanisms underlying the role of *MTSS1/*MIM-B in metastasis are needed, to facilitate the search for novel therapeutic strategies for HCC, and improve prognosis of patients with HCC undergoing hepatectomy.

## Abbreviations

AFP, α-fetoprotein; BBS, borate-buffered saline; ELISA, enzyme-linked immunosorbent assay; GEO, Gene expression omnibus; H&E, Hematoxylin and eosin; HCC, hepatocellular carcinoma; MIM, missing in metastasis; MMP2, matrix metalloproteinase 2; *MTSS1*, metastasis suppressor 1; PCR, polymerase chain reaction; PDGF, platelet derived growth factor; SDS-PAGE, sodium dodecyl sulfate-polyacrylamide gel electrophoresis; siRNA, small interfering RNA; SVM, Support vector machine; TACE, Transcatheter arterial chemoembolization; TGF-beta, transforming growth factor beta; VEGF, vascular endothelial growth factor.

## Additional file

Additional file 1:
**Figure S1**. The invasive ability (% of control) of MHCC97H cells. *A*, 0 h-24 h. *B*, 1 day (24 h)-35 day. Preliminary experiment was carried in 204 nude mice bearing HCC xenografts. 14 days after orthotopic implantation these mice were randomized into two groups (102 mice/group): palliative resection group and sham operation group (control). At 0 h, 1 h, 3 h, 5 h, 7 h, 9 h, 12 h, 18 h, 24 h (1 d), 3 d, 5 d, 7 d, 9 d, 14 d, 21 d, 28 d and 35 d after palliative resection, 6 mice from each group were humanely killed by cervical dislocation to harvest serum for cell culture studies. MHCC97H cells were treated with serum taken from above mentioned mice or cell culture medium supplemented with 10% human AB serum as control for 72 h, then these cells were added to the upper chamber (100 μL DMEM, 5 × 10^4^ cells/well) and 600 μL conditioned medium was added to the lower chamber. The invaded cells were fixed with methanol and stained with crystal violet solution after 24-hour incubation. The results were expressed as the number of penetrated cells under microscope at ×200 magnification on five random fields and were presented as means ± SD of three assays. MHCC97H cells treated with serum from the palliative resection group had the most invasive potential through Matrigel at 12 h and 14 d, as compared to cells in controls (*P* =0.027, 0.019, respectively). The bars indicate the means ± s.d. **Figure S2.** *MTSS1* promoted metastatic potential of Hep3B via transwell invasion assay. The number of penetrated Hep3B cells in the Vector-MTSS1 Group (*A*) was more than that in the Control Vector Group (*B*, *P* = 0.021). The assays were conducted in triplicate. (DOCX 3113 kb)
