# Erratum to: Elevated *MTSS1* expression associated with metastasis and poor prognosis of residual hepatitis B-related hepatocellular carcinoma

**DOI:** 10.1186/s13046-016-0373-4

**Published:** 2016-06-24

**Authors:** Xiu-Yan Huang, Zi-Li Huang, Bin Xu, Zi Chen, Thomas Joseph Re, Qi Zheng, Zhao-You Tang, Xin-Yu Huang

**Affiliations:** Department of General Surgery, Shanghai Jiaotong University Affiliated Sixth People’s Hospital, 600 Yi Shan Road, Shanghai, 200233 Peoples Republic of China; Department of Radiology, Xuhui Central Hospital, Shanghai, 200031 Peoples Republic of China; Department of General Surgery, The Tenth People’s Hospital of Tongji University, Shanghai, 200072 Peoples Republic of China; Thayer School of Engineering, Dartmouth College, Hanover, NH 03755 USA; Department of Radiology, Boston Children’s Hospital, Harvard Medical School, Boston, MA 02446 USA; Liver Cancer Institute and Zhongshan Hospital, Fudan University, Shanghai, 200032 Peoples Republic of China

## Erratum

Unfortunately, the original version of this article [1] did not include any funding information in the “Acknowledgements” section. The complete “Acknowledgements” section is included in full in this erratum.
