# Supplementary material for: Elevated MTSS1 expression associated with metastasis and poor prognosis of residual hepatitis B-related hepatocellular carcinoma
Source: J Exp Clin Cancer Res. 2016 May 26;35:85. doi: 10.1186/s13046-016-0361-8 (PMC4881066; doi:10.1186/s13046-016-0361-8)
Supplement: Additional file 1: — Figure S1. The invasive ability (% of control) of MHCC97H cells. A, 0 h-24 h. B, 1 day (24 h)-35 day. Preliminary experiment was carried in 204 nude mice bearing HCC xenografts. 14 days after orthotopic implantation these mice were randomized into two groups (102 mice/group): palliative resection group and sham operation group (control). At 0 h, 1 h, 3 h, 5 h, 7 h, 9 h, 12 h, 18 h, 24 h (1 d), 3 d, 5 d, 7 d, 9 d, 14 d, 21 d, 28 d and 35 d after palliative resection, 6 mice from each group were humanely killed by cervical dislocation to harvest serum for cell culture studies. MHCC97H cells were treated with serum taken from above mentioned mice or cell culture medium supplemented with 10% human AB serum as control for 72 h, then these cells were added to the upper chamber (100 μL DMEM, 5 × 10^4^ cells/well) and 600 μL conditioned medium was added to the lower chamber. The invaded cells were fixed with methanol and stained with crystal violet solution after 24-hour incubation. The results were expressed as the number of penetrated cells under microscope at ×200 magnification on five random fields and were presented as means ± SD of three assays. MHCC97H cells treated with serum from the palliative resection group had the most invasive potential through Matrigel at 12 h and 14 d, as compared to cells in controls (P =0.027, 0.019, respectively). The bars indicate the means ± s.d. Figure S2. MTSS1 promoted metastatic potential of Hep3B via transwell invasion assay. The number of penetrated Hep3B cells in the Vector-MTSS1 Group (A) was more than that in the Control Vector Group (B, P = 0.021). The assays were conducted in triplicate. (DOCX 3113 kb) [file 13046_2016_361_MOESM1_ESM.docx]

**Elevated *MTSS1* Expression Associated with Metastasis and Poor Prognosis of Residual Hepatitis B-related Hepatocellular Carcinoma**

**Xiu-Yan Huang^1@^**^*^**, Zi-Li Huang^2@^, Bin Xu^3@^, Zi Chen^4^, Thomas Joseph Re^5^, Qi Zheng^1^, Zhao-You Tang^6^, Xin-Yu Huang^1^**^*^

^1^Department of General Surgery, Shanghai Jiaotong University Affiliated Sixth People’s Hospital, Shanghai 200233, P.R. China.

^2^Department of Radiology, Xuhui Central Hospital, Shanghai 200031, PR China.

^3^Department of General Surgery, the Tenth People’s Hospital of Tongji University, Shanghai 200072, PR China.

^4^Thayer School of Engineering, Dartmouth College, Hanover, NH 03755, USA.

^5^Department of Radiology, Boston Children’s Hospital, Harvard Medical School, Boston, MA 02446, USA.

^6^Liver Cancer Institute and Zhongshan Hospital, Fudan University, Shanghai 200032, PR China.

**Corresponding authors:** Xiu-Yan Huang, MD, PhD, and Xin-Yu Huang, MD, Department of General Surgery, Shanghai Jiaotong University Affiliated Sixth People’s Hospital, 600 Yi Shan Road, Shanghai 200233, P.R. China. Tel & Fax: +86–21–64701361;

*E–mail: [xyhuang1119@163.com](mailto:xyhuang1119@163.com) [(X](mailto:qzheng818@yahoo.cn%20(QZ)-YH); [xinyuhuang9@163.com](mailto:xinyuhuang9@163.com) [(X](mailto:qzheng818@yahoo.cn%20(QZ)-YH)

**@**: These authors contributed equally to this work.

The authors have declared that no competing interests exist.





**Figure S1.** The invasive ability (% of control) of MHCC97H cells. *A*, 0 h-24 h. *B*, 1 day (24 h)-35 day. Preliminary experiment was carried in 204 nude mice bearing HCC xenografts. 14 days after orthotopic implantation these mice were randomized into two groups (102 mice/group): palliative resection group in which mice undergone partial HCC resection with the preservation of 2 mm tumor and sham operation group (control) in which mice undergone only exposure of the liver but no resection. At 0 h, 1 h, 3 h, 5 h, 7 h, 9 h, 12 h, 18 h, 24 h (1 d), 3 d, 5 d, 7 d, 9 d, 14 d, 21 d, 28 d and 35 d after palliative resection, 6 mice from each group were humanely killed by cervical dislocation to harvest serum for cell culture studies. MHCC97H cells were treated with serum taken from above mentioned mice or cell culture medium supplemented with 10% human AB serum as control for 72 hours, then these cells were added to the upper chamber (100 μL DMEM, 5 × 10^4^ cells/well) and 600 μL conditioned medium was added to the lower chamber. The invaded cells were fixed with methanol and stained with crystal violet solution after 24-hour incubation. The results were expressed as the number of penetrated cells under microscope at ×200 magnification on five random fields and were presented as means ± SD of three assays. MHCC97H cells treated with serum from the palliative resection group had the most invasive potential through Matrigel at 12 h and 14 d, as compared to cells in controls (*P* =0.027, 0.019, respectively), which was intervalidated by several HCC cell lines (HCCLM3, HCC7721, HCC7402, and Hep3B). The bars indicate the means ± s.d.

**Figure S2.** *MTSS1* promoted metastatic potential of Hep3B via transwell invasion assay. The number of penetrated Hep3B cells in the Vector-MTSS1 Group (*A*) was more than that in the Control Vector Group (*B*, *P* = 0.021). The assays were conducted in triplicate.
